# Ultrastructural examination of the corticocollicular pathway in the guinea pig: a study using electron microscopy, neural tracers, and GABA immunocytochemistry

**DOI:** 10.3389/fnana.2013.00013

**Published:** 2013-05-22

**Authors:** Kyle T. Nakamoto, Jeffrey G. Mellott, Jeanette Killius, Megan E. Storey-Workley, Colleen S. Sowick, Brett R. Schofield

**Affiliations:** Department of Anatomy and Neurobiology, Northeast Ohio Medical UniversityRootstown, OH, USA

**Keywords:** corticofugal pathways, inferior colliculi, auditory cortex, bouton classification, ultrastructural variations, synaptic targets

## Abstract

Projections from auditory cortex (AC) can alter the responses of cells in the inferior colliculus (IC) to sounds. Most IC cells show excitation and inhibition after stimulation of the AC. AC axons release glutamate and excite their targets, so inhibition is presumed to result from cortical activation of GABAergic IC cells that inhibit other IC cells via local projections. However, it is not known whether cortical axons contact GABAergic IC cells directly. We labeled corticocollicular axons by injecting fluorescent dextrans into the AC in guinea pigs. We visualized the tracer with diaminobenzidine and processed the tissue for electron microscopy. We identified presumptive GABAergic profiles with post-embedding anti-GABA immunogold histochemistry on ultrathin sections. We identified dextran-labeled cortical boutons in the IC and identified their postsynaptic targets according to morphology (e.g., spine, dendrite) and GABA-reactivity. Cortical synapses were observed in all IC subdivisions, but were comparatively rare in the central nucleus. Cortical boutons contain round vesicles and few mitochondria. They form asymmetric synapses with spines (most frequently), dendritic shafts and, least often, with cell bodies. Excitatory boutons in the IC can be classified as large, medium or small; most cortical boutons belong to the small excitatory class, while a minority (~14%) belong to the medium excitatory class. Approximately 4% of the cortical targets were GABA-positive; these included dendritic shafts, spines, and cell bodies. We conclude that the majority of cortical boutons contact non-GABAergic (i.e., excitatory) IC cells and a small proportion (4%) contact GABAergic cells. Given that most IC cells show inhibition (as well as excitation) after cortical stimulation, it is likely that the majority of cortically-driven inhibition in the IC results from cortical activation of a relatively small number of IC GABAergic cells that have extensive local axons.

## Introduction

The inferior colliculus (IC) is a large midbrain structure that integrates ascending information from the brainstem and descending information from cortical regions. It serves as the primary source of auditory projections to the thalamus (Saldaña et al., 1996; Winer et al., 1998; Malmierca, 2003; Malmierca and Hackett, 2010). The auditory cortex (AC) is the source of one of the largest inputs to the IC. Stimulation of the AC elicits excitation and inhibition in the IC in a variety of species (cat: Amato et al., 1969, 1970; Mitani et al., 1983; rat: Syka and Popelar, 1984; Popelar et al., 2003; guinea pig: Torterolo et al., 1998; mouse: Yan and Ehret, 2001; gerbil: Sakai and Suga, 2002; bat: reviewed in Suga, 2008). The excitation and inhibition can alter the responses of IC neurons for frequency, amplitude and duration tuning (Suga, 2008), rate-level functions (Popelar et al., 2003), pitch sensitivity (Nakamoto et al., 2010), spatial sensitivity (Jen et al., 1998; Nakamoto et al., 2008) and stimulus-specific adaptation (Anderson and Malmierca, 2013). While the majority of studies have measured the effects of AC stimulation on the ipsilateral IC, several studies have demonstrated that the contralateral IC is also affected (reviewed in Suga, 2008). The corticocollicular pathway has also been directly implicated in learning-induced auditory plasticity (Bajo et al., 2010). Despite the many functions attributed to this pathway, many questions remain about the underlying synaptic circuitry.

Mitani et al. (1983) showed that stimulation of the AC leads to short latency excitation of IC cells, followed by inhibition and, sometimes, by even later excitation. The initial excitation is presumably due to direct synaptic input from AC pyramidal cells, which are thought to be glutamatergic (Feliciano and Potashner, 1995). There is no evidence for GABAergic cortical projections to the IC, so the assumption is that AC axons activate GABAergic IC cells. Almost all IC cells project out of the IC and have axon collaterals that ramify within the IC. Consequently, cortical activation of IC GABAergic cells, which have local axon collaterals, could presumably account for cortically-driven inhibition of neighboring IC cells. GABAergic cells are present in all IC subdivisions (Merchán et al., 2005) and in many cases provide for GABAergic projections between IC subdivisions (González-Hernández et al., 1996; Hernández et al., 2006). Jen et al. (2001) provided evidence that AC projections activate cells in the lateral cortex of the IC (IClc) that project to the central nucleus of the IC (ICc). All of these ideas suggest that the AC activates IC GABAergic cells via direct synaptic contacts. However, there is no anatomical evidence for direct cortical inputs to IC GABAergic cells.

We used anatomical tracers and immunohistochemistry to label cortical axons and IC GABAergic cells. We applied multilabel electron microscopy to look for direct synaptic contacts between these elements. Our results provide evidence for such contacts, but suggest that they are rare compared to cortical synapses onto non-GABAergic IC cells. Our results also suggest that cortical boutons, generally considered to form a homogenous population, comprise two morphological types that are likely to differ in physiology and function.

## Experimental procedures

Experiments were performed on 5 adult pigmented guinea pigs of both genders weighing 400–900 g (Elm Hill Breeding Laboratories, Chelmsford, MA, USA). Additional data from these animals were used in a study for analysis of excitatory boutons in the IC (Nakamoto et al., 2013). All procedures were approved by the Institutional Animal Care and Use Committee (IACUC) and followed the National Institutes of Health guidelines for the care and use of laboratory animals. In accordance with these guidelines, all efforts were made to minimize the number of animals used and their suffering.

### Surgery

Each guinea pig was anesthetized prior to surgery with isoflurane (4–5% for induction, 1.75–3% for maintenance) in oxygen, or halothane (3.5% for induction, 2.5–2.75% for maintenance) in a mixture of oxygen and nitrous oxide. To reduce bronchial secretions, the animal was given atropine sulfate (0.08g mg/kg, i.m.). The animal's head was shaved and disinfected. A coating of antibiotic ointment (Neosporin Ophthalmic) was used to keep the eyes moist. A feedback-controlled heating pad was used to maintain body temperature of the animal. An incision was made in the scalp. A long-lasting local anesthetic (0.25% bupivacaine; Sensorcaine; Astra USA, Inc., Westborough, MA, USA) was injected into the margins of the incision. Surface landmarks (Bregma; pseudosylvian sulcus; Wallace et al., 2000, 2002) were used to guide all injections. The skull was opened at appropriate locations with a dental drill.

**Figure 1:**
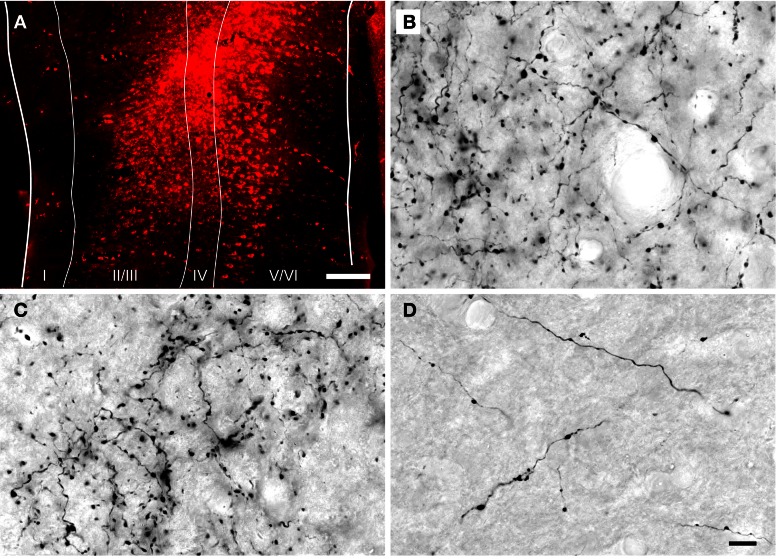
**Photomicrographs showing a representative injection into the auditory cortex and DAB-labeled cortical axons in the inferior colliculus. (A)** Transverse section through a FluoroRuby (FR) deposit site in the left auditory cortex. The white lines indicate cortical layers I through VI. **(B–D)** Photomicrographs of DAB-labeled cortical axons in the dorsal cortex **(B)**, lateral cortex **(C)** and central nucleus **(D)** of the inferior colliculus ipsilateral to an injection of FR in the auditory cortex. Labeled axons and boutons were observed in all subdivisions, but were much less numerous in the central nucleus. Parasagittal sections; rostral is to the right; dorsal is up. Scale bars = 200 μm for **(A)**, 20 μm for **(B–D)**.

Injections were made into the deep layers of the AC to label the corticocollicular pathway (Figure 1A). Two different fluorescent tracers were used: FluoroRuby (FR, 10% solution in saline; tetramethylrhodamine dextran, 10,000 molecular weight, Invitrogen, Eugene, OR) or fluorescein dextran (FD, 10% in saline, molecular weight = 10,000, Invitrogen, Eugene, OR). Each tracer was injected with a microsyringe dedicated for use only with that tracer. Two methods were used to deposit tracer in the AC. For the first method, a 10 μl microsyringe was inserted into the cortex dorsomedial to the AC and advanced ventro-laterally through the AC. Multiple injections of 0.2 μl of FD or FR were made during the withdrawal of the microsyringe. The syringe was inserted 2–3 times along the rostral-caudal extent of the AC, for a total injection volume of 4–6 μl of tracer. In the second method, 5–37 injections were made with a 10 μl Hamilton microsyringe oriented perpendicular to the cortical surface. A total 1.0–7.4 μl of tracer was injected ventrolateral to the pseudosylvian sulcus and 1–6 mm caudal to Bregma. This region is centered on the core AC areas (Wallace et al., 2000). Upon completion of the injections, the exposed brain was covered with Gelfoam and the scalp was sutured.

### Perfusion and sectioning

After 11–19 days to allow for axonal transport, animals were individually sacrificed by overdose with sodium pentobarbital (440 mg/kg; i.p., Euthasol, Virbac Inc., Fort Worth, TX, USA) or isoflurane (inhalation until cessation of breathing; Aerrane, Baxter, Deerfield, IL, USA). Immediately after cessation of breathing the animal was perfused through the aorta with Tyrode's solution, followed by 2% paraformaldehyde and 2% glutaraldehyde in 0.1 M phosphate buffer (PB, pH 7.4). The brain was then removed and stored overnight at 4°C in 2% paraformaldehyde and 2% glutaraldehyde in PB. The following day, a Vibratome was used to cut 50 μm parasagittal sections from tissue blocks containing the IC. Six series of sections were collected and processed as described below or stored in freezing buffer at −20°C for future processing.

### Tissue preparation

An antibody for FR or FD was used to label cortical boutons in the IC. Sections were treated with 10% normal goat serum (NGS) with 0.3% Triton X-100 in phosphate buffered saline (PBS) (0.9% NaCl in 0.01 M phosphate buffer, pH 7.4) for 1 h (all steps at room temperature unless noted). Goat biotinylated anti-rhodamine (Vector Laboratories Cat. No BA-0605) or goat biotinylated anti-fluorescein (Vector Laboratories Cat. No BA-0601) was applied with 1% NGS in PBS overnight at 4°C. The concentration of antibody varied from 1:400 to 1:1000. Following three 5-min washes in PBS, the sections were incubated in avidin–biotin–peroxidase (ABC Elite kit, Vector Laboratories), then rinsed and stained with diaminobenzidine (DAB) with nickel enhancement (Adams, 1981). Sections to be examined with the light microscope were mounted on slides, dried overnight and then coverslipped with DPX (Aldrich Chemical Company, Inc., Milwaukee, WI, USA). Sections to be examined with electron microscopy were processed further, as described below.

One series of sections from each animal was stained for nicotinamide adenine dinucleotide phosphate diaphorase (NADPH) activity (Dawson et al., 1991). The stained sections were mounted on slides, dried overnight and then coverslipped with DPX (Aldrich Chemical Company, Inc., Milwaukee, WI, USA). The NADPH stain reflects the distribution of neuronal nitric oxide synthase and can be used to distinguish ICc from the surrounding subdivisions (Coote and Rees, 2008; detailed in Nakamoto et al., 2013).

### Processing for electron microscopy

**Figure 2:**
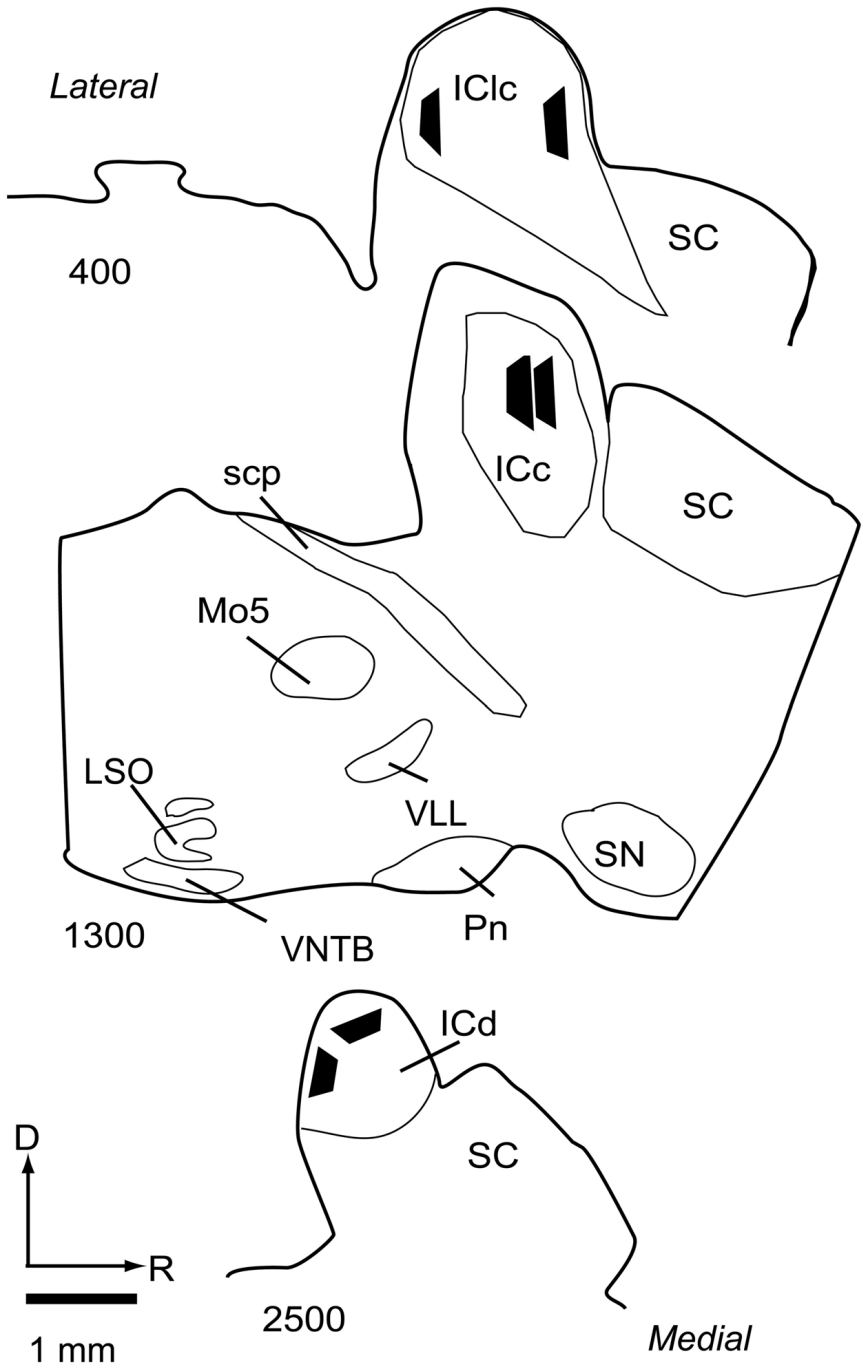
**Line drawings of parasagittal sections through the IC showing the locations of the 6 tissue blocks collected for analysis.** Two blocks were collected from each of 3 subdivisions of the IC. Each black trapezoid represents the position of a single tissue block. Samples were taken from multiple sections across several animals, but are drawn on a single representative series of sections for ease of comparison. Sections are arranged from lateral to medial; the numbers at lower left of each section indicate approximate distance of the section, in μm, from the lateral-most section in the series. Abbreviations: D, dorsal; ICc, central nucleus of the inferior colliculus; ICd, dorsal cortex of the inferior colliculus; IClc, lateral cortex of the inferior colliculus; LSO, lateral superior olive; Mo5, motor trigeminal nucleus; Pn, pontine nuclei; R, rostral; SC, superior colliculus; scp, superior cerebellar peduncle; SN, substantia nigra; VLL, ventral nucleus of the lateral lemniscus; VNTB, ventral nucleus of the trapezoid body.

Selected sections containing the IC (not stained for NADPH) were post-fixed for 1 h in 2% osmium tetroxide in PB, dehydrated in an alcohol series, embedded in Durcupan resin (Electron Microscopy Sciences, Fort Washington, PA, USA) and flat-mounted between sheets of Aclar Embedding Film (Ted Pella, Inc., Redding, CA, USA). The sections were from the IC ipsilateral to the cortical injections, because the ipsilateral IC receives substantially more cortical input than the contralateral IC (Coomes et al., 2005). The sections were examined in a light microscope (Zeiss AxioImager Z1) to identify areas that contained a large number of labeled cortical boutons. These regions were then compared to NADPH-stained sections to identify IC subdivisions. An area that was completely contained within an IC subdivision was trimmed from the section with a scalpel and glued onto a resin base with cyanoacrylate (KrazyGlue, Columbus, OH, USA). A Zeiss Axioplan 2 microscope with a Neurolucida system (MBF Bioscience, Williston, VT) was used to draw the IC section and plot the position of the trimmed area. Borders of the IC subdivisions were added by superimposing the image of a nearby section stained for NADPH. For illustration purposes, the location of each area was transferred to a single representative series of sections (Figure 2).

An ultramicrotome (UC6 Ultramicrotome, Leica Microsystems, Buffalo Grove, IL, USA) was used to cut ultrathin sections (100 nm, gold-silver interference color) from each tissue block. Every seventh section was collected (this spacing is greater than the dimensions of synaptic zones in the IC, ensuring that a single synapse was not included in more than one ultrathin section for the quantitative analyses; Nakamoto et al., 2013). Individual sections were collected on 300-mesh nickel grids and immunostained for GABA, as described previously (Coomes et al., 2002). Grids with ultrathin sections were incubated overnight in anti-GABA antibody (rabbit anti-GABA, Sigma, St. Louis, MO) diluted 1:500 or 1:1000 in 0.05 M Tris-buffered saline with 0.1% Triton X-100 (TBST) pH 7.6, washed in TBST pH 7.6, then TBST pH 8.2, and placed into a secondary antibody conjugated to 15 nm gold particles (goat anti-rabbit, diluted 1:25 in TBST pH 8.2; Ted Pella Inc., Redding, CA). The sections were washed in TBST pH 7.6, washed in water, stained with uranyl acetate (1% aqueous) or uranyl acetate and Reynolds's lead citrate (Reynolds, 1963), and allowed to dry.

### Electron microscopy and image preparation

Ultrastructure was observed with a transmission electron microscope (JEM-100S; JEOL, Peabody, MA, USA) at 60kV and 15,000–40,000 magnification. Images were recorded on Kodak SO-163 film (Kodak, Rochester, NY. USA). The negatives were scanned at a resolution of 1200–2000 pixels/inch (ScanMaker 800, Microtek, Santa Fe Springs, CA, USA) to produce digital images for analysis. Profiles and vesicles in the image were analyzed with ImageJ (Abramoff et al., 2004). Adobe Illustrator and Adobe Photoshop (Adobe, San Jose, CA, USA) were used to adjust brightness and contrast levels, to arrange and label photographs, and to add colors to facilitate descriptions. Graphs were generated with Excel (Microsoft Corporation, Redmond, WA, USA).

### Identification of cortical synapses

**Figure 3:**
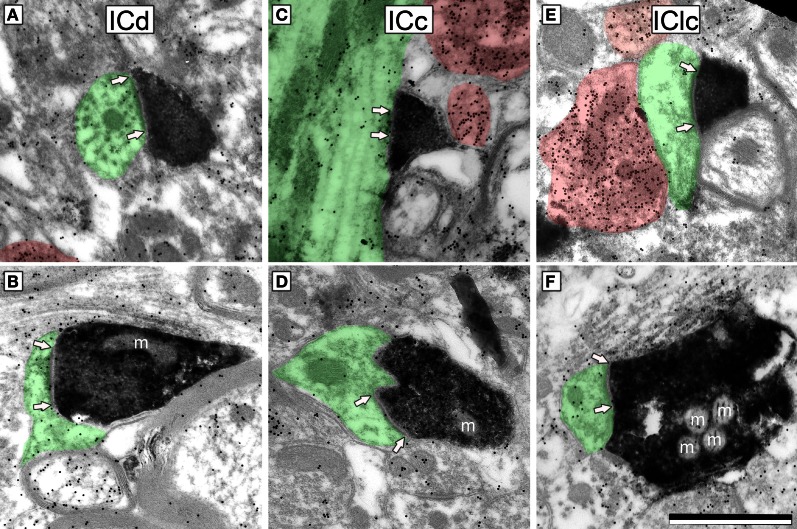
**Electron micrographs showing examples of DAB-labeled cortical boutons in the IC.** Presynaptic cortical boutons are labeled with DAB (electron dense cytoplasm) and postsynaptic profiles are shaded green. Arrows bracket the synaptic densities and are placed on the postsynaptic profile. GABA immunoreactivity is demonstrated by the density of gold particles (black dots; GABA-immunopositive profiles are highlighted by red shading). Cortical boutons have prominent postsynaptic densities, are filled with round synaptic vesicles, and are GABA-negative (compare with the GABA-positive profiles). Cortical boutons were found in ICd **(A,B)**, ICc **(C,D)** and IClc **(E,F)**. m, mitochondria. Scale bar = 1 μm.

We identified cortical synapses by the pre-synaptic DAB label, the presence of a post-synaptic density (Figure 3, bracketed with arrows), a synaptic cleft, a collection of vesicles in the presynaptic profile (Figure 3) and a post-synaptic profile (Figure 3, shaded green). For both ICd and IClc, sections were systematically scanned and care was taken to photograph every DAB profile that formed a synapse. Labeled boutons were comparatively rare in the ICc, so we did not quantify this group.

### Identification of GABA immunoreactivity

In a previous study that used the same GABA antibody and staining protocol, the density of gold particles over a profile was analyzed quantitatively and compared to staining in surrounding tissues (Coomes et al., 2002). The staining was sufficiently robust that simple visual inspection proved as reliable as the quantification for determining whether a profile was immunopositive (similar to visual classification of profiles as DAB-labeled vs. unlabeled). In the present study, the GABA immunostaining was equally robust and the GABA-positive (Figure 3, red) profiles were assessed by visual comparison of the number of gold particles over a profile relative to background labeling. As with any immunostaining procedure, background staining can vary across sections and across cases, so all assessments were done by comparison with nearby regions.

## Results

Anterograde tracer injections were centered in the primary AC and, in most cases, extended into the surrounding areas [e.g., the dorsocaudal field, ventrorostral and dorsorostral belt areas, as defined by Wallace et al. (2000)]. The injections extended to the deep layers of AC (Figure 1A), which include cells that project to the IC (Kelly and Wong, 1981; Coomes et al., 2005; Schofield, 2009). Anterograde labeling was present in many areas known to receive AC projections, including the medial geniculate body (ipsilaterally) and, bilaterally, in the IC, superior olivary complex and cochlear nucleus. Figures 1B–D shows representative labeling of axons and boutons in the ipsilateral IC. Retrograde labeling was visible throughout the medial geniculate body. These labeling patterns are consistent with a large AC injection (Redies et al., 1989).

### Cortical boutons belong to 2 morphological types of excitatory boutons in the IC

For quantitative description, we used a sample of 52 cortical synapses in the IClc and 51 cortical synapses in the ICd. Cortical synapses appeared similar in all areas examined (Figure 3). Cortical boutons almost always contained densely-packed round vesicles. They formed synapses with substantial post-synaptic densities, suggestive of asymmetric synapses (although this could not be observed directly because the DAB reaction product in the cortical bouton obscured the presynaptic density). Almost all (>99%) cortical boutons were GABA-negative.

We quantified several characteristics of the labeled boutons in order to relate them to our previous classification of excitatory synapses in the IC (Nakamoto et al., 2013). Most (86%) of the labeled cortical boutons are less than 0.7 μm^2^ in area and contain 1 or no mitochondria (e.g., Figures 3A–E, mitochondria are indicated by an “m”). These boutons belong to the small excitatory (SE) class of IC boutons. The remaining labeled boutons (14%) are larger than SE boutons (0.76–1.13 μm^2^ in area) and contain 2–4 mitochondrial profiles. These boutons belong to the medium excitatory (ME) class (e.g., Figure 3F). None of the labeled boutons are characteristic of the third class of excitatory boutons with large excitatory morphology.

### Cortical boutons most often contact GABA-negative spines

**Figure 4:**
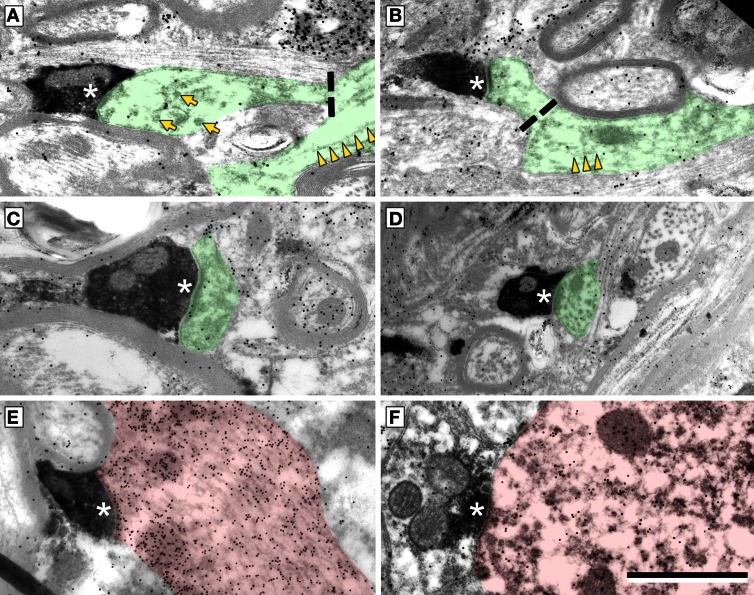
Electron micrographs showing examples of electron dense cortical boutons forming synapses (^*^) onto dendritic spines (A–C), dendritic shafts (D,E) or a soma (F). (A,B) Synapses onto GABA-negative dendritic spines with parent dendrites visible. Dashed line indicates the junction of the spine neck and dendritic shaft. Microtubules (yellow arrowheads) are present in dendrites, but not in spines. Smooth walled sacs (yellow arrows) are present in some spines. **(C)** Synapse from a cortical bouton onto a spine without a visible parent dendrite. **(D)** Synapse onto a dendritic shaft, identified by microtubules in the postsynaptic profile. **(E,F)** Synapses onto a GABA-positive (red) dendritic shaft **(E)** and a GABA-positive (red) soma **(F)**. Scale bar = 1 μm.

**Figure 5:**
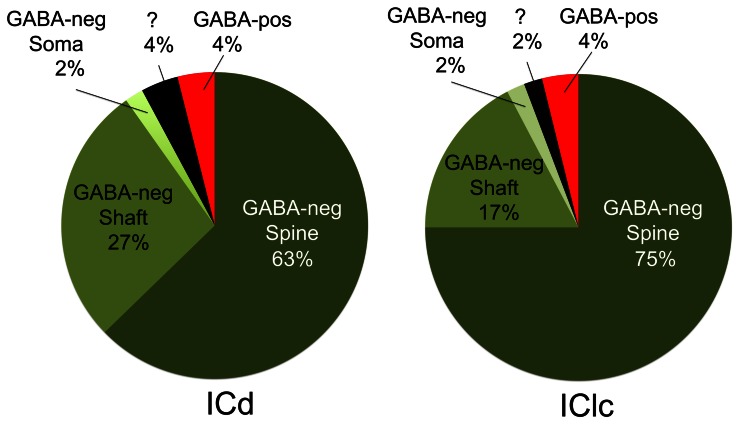
**Pie charts showing the frequency with which cortical boutons contact GABA-negative dendritic spines, GABA-negative dendritic shafts, GABA-negative somas and GABA-positive profiles.** In both ICd and IClc, GABA-negative dendritic spines were the most frequent targets of cortical boutons, followed by GABA-negative dendritic shafts. GABA-positive targets included spines, dendritic shafts and somas, but were so rare that they are represented as a single group. A few post-synaptic targets could not be identified with certainty and are shown with a question mark. Abbreviations: ICd, dorsal cortex of the inferior colliculus; IClc, lateral cortex of the inferior colliculus.

Cortical boutons target both GABA-negative profiles (Figure 4, shaded green) and GABA-positive profiles (Figure 4, shaded red). In both the ICd and the IClc, GABA-negative profiles were contacted far more often (92–94% of targets) than GABA-positive profiles (4% of targets). Among the GABA-negative targets, spines were contacted most often, followed by dendritic shafts and somas (Figure 5). In general, the results were similar between ICd and IClc, except that targets were biased more heavily toward spines in the IClc. The relative occurrence of different targets appeared similar for SE and ME boutons (after accounting for the larger number of SE boutons overall). Of 71 cortical contacts onto spines, 61 were from SE boutons and 10 from ME boutons (recall that ME boutons constituted 14% of the total labeled boutons). Contacts onto GABA-negative dendritic shafts also included both SE and ME boutons (21 SE, 2 ME). The 2 synapses onto GABA-negative somas were made by SE boutons.

GABA-positive profiles accounted for 4% of the cortical targets (Figure 5). Despite the small number of such synapses in our sample, the targets included dendrites, spines and somas. The boutons included members of the SE class and a single example from the ME class (contacting a soma).

## Discussion

The present study demonstrates that cortical boutons contact GABA-negative and GABA-positive profiles in the IC, and provides a quantitative description of cortical boutons in the guinea pig IC. The majority of cortical boutons belong to the small excitatory (SE) class, with the remaining boutons belonging to the medium excitatory (ME) class. The morphological features suggest that the two classes of boutons may have different cellular origins and may serve different functions. The targets include spines, dendritic shafts and, least often, somas. Cortical contacts onto GABAergic cells presumably underlie at least some of the cortically-driven inhibition of IC cells.

### Technical considerations

The anti-GABA antibody used in the present study has been used in numerous previous studies (Coomes et al., 2002; Ruiz et al., 2004; Ponti et al., 2008; Luzzati et al., 2009; Brown et al., 2012; Smith et al., 2012; Nakamoto et al., 2013), and there appear to be few concerns about the specificity of this GABA antibody. The ultrastructural features of GABA-positive profiles labeled in the present study are similar to other descriptions of GABAergic profiles in the IC (Roberts and Ribak, 1987; Oliver and Beckius, 1992). A second concern with any immunostain is sensitivity. We chose a concentration of primary antibody that labels “expected” profiles (e.g., boutons with pleomorphic vesicles) as well as a subset of somas, dendrites, spines, and axons, consistent with results from other studies and other techniques. At the chosen concentration, the antibody almost never labeled “unexpected” profiles, such as presynaptic boutons with round vesicles. Tests in which a higher concentration of primary anti-GABA was used (as high as 1:100 dilution; compared to our “usual” working solution of 1:1000), *did* yield excessive label, including labeling of many boutons with round vesicles and asymmetric synapses. We conclude that our immunostaining is likely to be specific, but it remains possible that some GABAergic profiles went unstained. Such a result would lead us to underestimate the percentage of GABAergic targets of AC boutons.

There are also limitations associated with the tracers. We made large injections to maximize the number of labeled axons and thus minimize the chances of missing connections. However, none of our cases had injections that spanned the entire AC. In particular, the more caudal regions, especially the belt areas around the caudal end of the dorsocaudal field (c.f. Wallace et al., 2000, 2002), were not likely to have been involved in any of our injections. We would not be surprised to discover differences in projections from different cortical areas, but such information will have to be based on future studies with smaller injections. Despite these limitations, our observations are generally similar to those from previous studies. The cortical axons terminated densely in the ICd and IClc, and less densely in the ICc, in agreement with previous reports based on anterograde tracing (Feliciano and Potashner, 1995; Winer et al., 1998; Bajo and Moore, 2005; Bajo et al., 2007) and our previous studies with retrograde tracers in guinea pigs (Coomes et al., 2005; Schofield, 2009).

### Implications of cortical bouton ultrastructure

The cortical boutons contain round vesicles and have prominent post-synaptic densities, similar to those described in rats and cats (Jones and Rockel, 1973; Granstrem, 1984; Saldaña et al., 1996). We also found that the cortical boutons are GABA-negative. All these features suggest an excitatory role for corticocollicular axons. Anatomical studies in guinea pigs suggest that corticocollicular cells use glutamate (Feliciano and Potashner, 1995; Saint Marie, 1996) and physiological studies using electrical stimulation of the AC during intracellular recording of IC cells show evidence for monosynaptic excitation (Mitani et al., 1983).

We recently classified presumptive excitatory synapses—GABA-negative boutons that contain round vesicles and form asymmetric synapses—in the guinea pig IC (Nakamoto et al., 2013). Three classes can be distinguished on the basis of bouton profile area and number of mitochondrial profiles. Large excitatory (LE) boutons contain many mitochondrial profiles (generally more than 4, and up to 20 or more). Small excitatory (SE) boutons usually contain 0 or 1 mitochondrial profiles in a single thin section. Medium excitatory (ME) boutons contain an intermediate number of mitochondria. This classification scheme provides a basis for categorizing the corticocollicular synapses in the present study.

The majority of cortical boutons belong to the SE class (86%). The majority of excitatory boutons in ICd (80%) and IClc (86%) belong to the SE class (Nakamoto et al., 2013). In other brain areas, small boutons have been associated with weaker post-synaptic effects compared to larger boutons (Pierce and Lewin, 1994), suggesting that *individual* cortical boutons may have a relatively small effect in the IC. The presence of mitochondria has been associated with high metabolic activity and the ability of a synapse to sustain high rates of firing and use reserve vesicles pools (Nguyen et al., 1997; Ly and Verstreken, 2006). The scarcity of mitochondria in SE cortical boutons suggests that they do not routinely sustain high rates of firing.

A minority of cortical boutons are of the ME type (14%). ME boutons are likely to serve a different function. A minority of excitatory boutons in ICd (20%) and IClc (12%) belong to the ME class (Nakamoto et al., 2013). Both SE and ME boutons were labeled even when the injection site was limited to A1, so it is not the case that the different bouton types arise from different cortical areas. While it is possible that a single cortical cell could give rise to both bouton types, we suggest that the two bouton types arise from different classes of cortical cells. One possibility is that the SE boutons arise from layer V pyramidal cells and ME boutons arise from layer VI cells. Layer VI cells make up only about 10% of the corticocollicular cells, and thus would be expected to provide only a minority of labeled boutons in our cases (Schofield, 2009). This possibility appears unlikely because layer VI projects to ICd and IClc but not to ICc (Schofield, 2009), but even our small sample of boutons in the ICc included examples of both ME and SE boutons. A more likely possibility is that SE and ME boutons arise from different types of pyramidal cell in layer V. Bajo and Moore (2005) suggested that two morphological types of layer V pyramidal cell project to the IC in gerbils. Lu et al. (2007) provided evidence in guinea pigs for two classes of layer V corticocollicular cells that differ both in morphology and intrinsic physiology. Large cells with a substantial apical dendritic tuft showed bursting responses to a current injection, whereas smaller cells lacked a tuft and had regular spiking responses to current injection. These two types of layer V pyramidal cells may have different bouton morphology in the IC. One can speculate that the bursting nature of the tufted cells might be more consistent with the ME bouton type, with mitochondrial support for bursting activity. It follows that non-bursting, “regular-spiking” cells may then have SE boutons.

Both SE and ME classes include boutons that have densely-packed vesicles and boutons that have loosely-packed vesicles (Nakamoto et al., 2013). Almost all the cortical boutons have densely packed vesicles, suggesting that boutons with loosely-packed vesicles originate from non-cortical sources. The ICd and IClc (which contain many ME boutons and most of the SE boutons) receive relatively little input from the lateral lemniscus (reviewed in Winer and Schreiner, 2005; Malmierca and Hackett, 2010), suggesting that lower auditory nuclei are not major sources of the boutons with loosely-packed vesicles. The only other major source of excitatory boutons in the IC is from IC cells themselves (reviewed in Saldaña and Merchan, 2005). This would include local collaterals from cells in the same IC and/or commissural projections from cells in the contralateral IC. Additional experiments will be needed to determine whether IC cells are the major source of SE and ME boutons that have loosely-packed vesicles.

Do densely-packed vesicles serve to identify cortical boutons? This almost certainly is not the case for ME boutons. Unlike cortical boutons, ME boutons are quite numerous in the ICc, and many are likely to originate from lemniscal sources (Nakamoto et al., 2013). Whether AC is the sole source of SE boutons with densely packed vesicles is harder to determine. SE boutons overall are distributed similarly to cortical boutons (i.e., they are most numerous in ICd and IClc and less numerous in ICc). While we observed SE boutons with densely packed vesicles that were not labeled with tracer, it is impossible to determine whether these originated from cortical cells that were not labeled by our injections or from non-cortical sources. This issue may be best addressed by additional studies to see if such boutons are labeled in association with other pathways.

### Subcellular targets of cortical boutons

The present data demonstrate cortical synapses on dendritic spines, dendritic shafts and somas. Although the somatic contacts are the least common, they are of interest because their proximity to the presumed action potential trigger zone suggests that they may have a particularly powerful effect on the target cell. Nonetheless, the majority of cortical synapses in the present study contacted dendritic shafts and spines, similar to descriptions of cortical synapses in cats (Jones and Rockel, 1973; Granstrem, 1984). We noted a higher percentage of contacts onto spines (ICd: 63%; IClc: 75%) than described in rats (ICd: 28%, IClc: 27%; Saldaña et al., 1996). While the differences may reflect the different species, they may also be due in part to a difference in criteria for identifying spines. Saldaña et al., (1996) used the presence of mitochondria as a marker for dendritic shafts. We have found that spines in guinea pig IC, like those in cat IC, can contain mitochondria (Rockel and Jones, 1973; Nakamoto et al., 2013), which is unusual for simple spines (Chicurel and Harris, 1992). As Saldaña et al., (1996) noted, the termination on spines and small dendrites is consistent with a modulatory role for the cortical boutons. Targeting of spines is also consistent with a role in synaptic plasticity (Nimchinsky et al., 2002; Harris, 2003; Roberts et al., 2010) and numerous physiological and behavioral studies that have linked the corticocollicular projection to auditory plasticity (Bajo et al., 2010; Suga, 2012).

### GABAergic vs. non-GABAergic targets of cortical boutons

**Figure 6:**
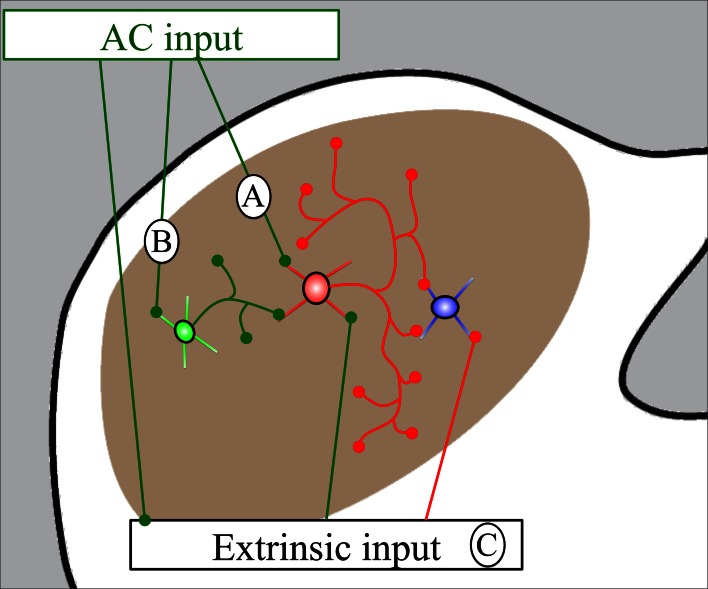
**Schematic diagram showing routes by which auditory cortical (AC) stimulation could generate inhibition in the IC. (A)** Cortical stimulation activates GABAergic cells (red) that have extensive local collaterals and inhibit subsequent IC cells (blue). **(B)** Cortical stimulation activates glutamatergic cells (green) that activate GABAergic cells (red) which inhibit subsequent cells (blue). **(C)** Cortical stimulation activates cells extrinsic to the IC that directly or indirectly inhibit IC cells. This diagram is not subdivision specific and these effects could occur within or between the IC subdivisions.

We observed cortical synapses with both GABA-positive and GABA-negative IC cells. The majority of IC cells are considered to be either GABAergic or glutamatergic, so it is reasonable to assume that most GABA-negative IC cells are likely to be glutamatergic (reviewed in Kelly and Caspary, 2005). Our findings are consistent with predictions from physiological studies in which ipsilateral IC cells generally showed early excitation followed by inhibition after stimulation of the AC (Mitani et al., 1983; Syka and Popelar, 1984; Torterolo et al., 1998; Suga, 2012). The corticocollicular cells are believed to be glutamatergic and excitatory (Feliciano and Potashner, 1995), and presumably account for early, monosynaptic cortical excitation of IC cells. Cortical inhibition of IC cells was thought to result from AC activation of IC GABAergic cells that inhibit other IC cells through local connections (either within or between IC subdivisions; Jen et al., 2001). The present study is the first to use double-label electron microscopy to look for AC contacts on IC GABAergic cells. Our previous study demonstrated that about 11–12% of excitatory boutons in the ICd and IClc contact GABAergic cells (Nakamoto et al., 2013). Given the physiological studies discussed above, we expected many cortical boutons to contact GABAergic cells. While we observed such contacts in both the ICd and the IClc, we were surprised at how rarely this occurred; less than 5% of cortical synapses in either area were formed with GABA-positive targets. The simplest explanation for the initial inhibition that occurs after AC stimulation is that the small number of IC GABAergic cells contacted by cortex have extensive local axons, contacting many IC cells (Figure 6A). This is consistent with the short latency of the initial inhibition that occurs after cortical stimulation (Mitani et al., 1983). The conclusion that cortically-targeted IC GABAergic cells have extensive intracollicular connections is consistent with observations that almost all IC cells have extensive local axon collaterals (e.g., Oliver, 2005; Saldaña and Merchan, 2005) and highlights the functional significance of inhibitory circuits within the IC. The short latency of the initial inhibition argues against longer chains of connections, such as AC activation of glutamatergic IC cells that subsequently activate inhibitory cells (Figure 6B) and/or AC activation of cells extrinsic to the IC (e.g., periolivary, cochlear nucleus) that subsequently inhibit IC cells (Figure 6C). However, there can be multiple waves of inhibition in the IC after cortical stimulation, and it seems likely that the longer chains of connections contribute to this later inhibition.

### Cortical inputs and IC outputs

The IC projects to many targets, and evidence is growing that cortical inputs influence some of these pathways by direct synaptic inputs. Previous studies with light microscopy suggested that cortical axons contact IC cells that project to the MG or to the CN (Schofield and Coomes, 2006; Coomes Peterson and Schofield, 2007). Both of these studies provided evidence for cortical inputs to IC somas, dendritic shafts and dendritic spines. The present study confirms that such corticocollicular synapses exist. Preliminary studies also suggest that cortical axons directly contact commissural cells, the source of the large pathway connecting left and right IC (Nakamoto and Schofield, 2009). While GABAergic cells are thought to contribute to a number of these pathways (namely, the colliculogeniculate and commissural pathways), glutamatergic cells are predominant in the IC and its outputs.

### Conflict of interest statement

The authors declare that the research was conducted in the absence of any commercial or financial relationships that could be construed as a potential conflict of interest.

## Abbreviations

AC: auditory cortex
D: dorsal
DAB: diaminobenzidine
FD: fluorescein dextran
FR: FluoroRuby
GABA-negative: gamma-aminobutyric acid immunonegative
GABA-positive: gamma-aminobutyric acid immunopositive
IC: inferior colliculus
ICc: central nucleus of the inferior colliculus
ICd: dorsal cortex of the inferior colliculus
IClc: lateral cortex of the inferior colliculus
LE: large excitatory bouton type
LSO: lateral superior olive
m: mitochondria
ME: medium excitatory bouton type
Mo5: motor trigeminal nucleus
NADPH: nicotinamide adenine dinucleotide phosphate
NGS: normal goat serum
PB: 0.l M phosphate buffer
PBS: phosphate buffered saline
Pn: pontine nuclei
R: rostral
SC: superior colliculus
scp: superior cerebellar peduncle
SE: small excitatory bouton type
SN: substantia nigra
TBST: tris-buffered saline with 0.1% triton X-100
VLL: ventral nucleus of the lateral lemniscus
VNTB: ventral nucleus of the trapezoid body.
